# Wireless and Powerless Sensing Node System Developed for Monitoring Motors

**DOI:** 10.3390/s8085005

**Published:** 2008-08-27

**Authors:** Dasheng Lee

**Affiliations:** Department of Energy and Refrigerating Air-conditioning Engineering, National Taipei University of Technology, Taipei, Taiwan, 106; E-Mail: f11167@ntut.edu.tw; Tel.: +886-2-2771-2171; Fax: +886-2-2731-4919

**Keywords:** Sensor networks, motor monitoring system, wireless and powerless sensing node, EM pulse, monitoring system reliability

## Abstract

Reliability and maintainability of tooling systems can be improved through condition monitoring of motors. However, it is difficult to deploy sensor nodes due to the harsh environment of industrial plants. Sensor cables are easily damaged, which renders the monitoring system deployed to assure the machine's reliability itself unreliable. A wireless and powerless sensing node integrated with a MEMS (Micro Electro-Mechanical System) sensor, a signal processor, a communication module, and a self-powered generator was developed in this study for implementation of an easily mounted network sensor for monitoring motors. A specially designed communication module transmits a sequence of electromagnetic (EM) pulses in response to the sensor signals. The EM pulses can penetrate through the machine's metal case and delivers signals from the sensor inside the motor to the external data acquisition center. By using induction power, which is generated by the motor's shaft rotation, the sensor node is self-sustaining; therefore, no power line is required. A monitoring system, equipped with novel sensing nodes, was constructed to test its performance. The test results illustrate that, the novel sensing node developed in this study can effectively enhance the reliability of the motor monitoring system and it is expected to be a valuable technology, which will be available to the plant for implementation in a reliable motor management program.

## 1. Introduction

Condition monitoring techniques have been researched and developed for several years. Through the monitoring of the health of running electric motors, severe economic losses resulting from unexpected motor failures can be avoided and tooling system reliability and maintainability can be improved [1-4]. Identifying mechanical and electrical maintenance problems with sensor feedback data and correcting them will reduce unplanned production shutdowns and significantly increase profits [5]. In addition to the obvious benefits for industrial manufacturers, a system incorporating energy usage evaluations and motor condition monitoring functions has also been proposed for industrial emissions reduction. It is anticipated by the U.S. Department of Energy (DOE) that by 2010 the widespread deployment of sensor networks in industrial monitoring systems could improve overall production efficiency by 11% to 18%, and in addition, reduce industrial emissions by more than 25% [6].

Wireless communication techniques enable a new class of low cost and flexible condition monitoring systems. Traditional monitoring systems in industrial plants are realized through wired systems, formed by communication cables and sensors [7-9]. The installation and maintenance of these cables and sensors is much more expensive than the cost of the sensors themselves. Integration of electronics, sensors and wireless communications has enabled the easy installation of these sensor networks, which saves the cost of the deployment of large numbers of sensors and actuators [10]. The flexibility and rapid deployment characteristics of a wireless sensor network (WSN) form an ideal platform for condition monitoring systems. Although wireless communication has enabled WSN with applications for condition monitoring, sensing nodes still require an electric power supply for operations and their power line distribution conflicts with ease of installation and retrofit into industrial plants. Furthermore, the sensor cables are easily damaged, which affects the reliability of the monitoring system deployed to assure the machine is reliable. In this study, a sensing node was developed to achieve wireless and powerless operation for implementing an easily mounted sensor networks for monitoring motor conditions. A wireless-equipped monitoring system and powerless sensing nodes was constructed for tests and it is expected to be a reliable machine management program.

## 2. Methods

### 2.1. Wireless and powerless sensing node

The design of the wireless and powerless sensing node provides a solution that can work independently inside a motor. A specially designed communication module transmits electromagnetic (EM) pulses in response to a sensor output, and the pulses are able to pass through the motor casing to deliver the signal to the data acquisition terminal. No signal cable passing through the motor case is required, and as induction power is generated from the motor shaft rotation, the resulting sensing system is self-sustaining and no power lines are required.

**Figure 1.**
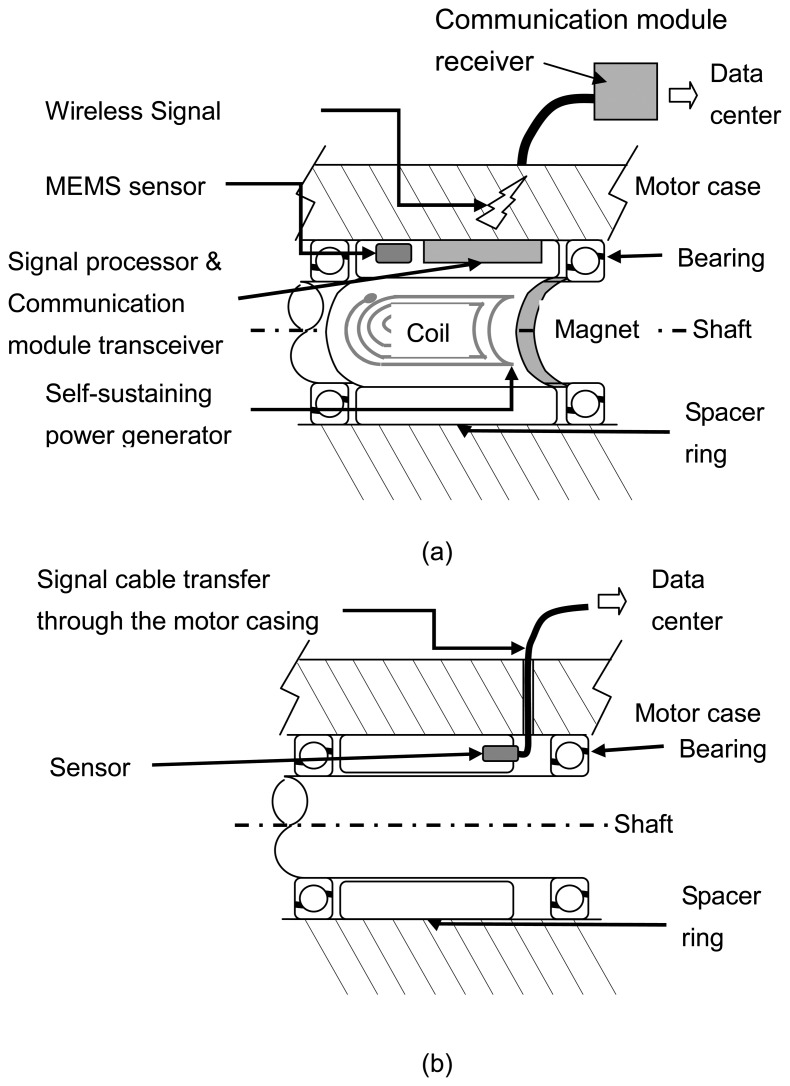
The schematic views of **(a)** Condition monitoring system with wireless and powerless sensing node; **(b)** Traditional system.

Figure 1(a) shows the schematic view of the sensing node. A sensor, a signal processor, a communication module, and a magnetic self-powered generator are integrated into the spacer ring. The spacer is fit into the shaft, located between two bearing sets in the motor. Four planar coils, distributed around the ring generate power by the induction of the coils and soft magnets are attached to the shaft as the motor starts. The induction power drives the sensor, a signal processor, and the communication module.

Through coil arrangements and circuit design, the sensing node on the ring is self-sustaining. The communication module antenna, fitted tightly into the spacer ring inside the motor, is in direct contact with the inner surface of the motor case. EM pulses, transmitted from the module delivery sensor signal, go to the outside data acquisition center through the motor casing. The signal cable attached on the motor's surface works as a receiver antenna and transmits the signal to the data acquisition center. The special wireless signal transfer uses the metal wall as the media to transmit data between sensors and the data center. No physical signal cable enters through the metal casing of the motor. Only one signal cable, which is attached on the outside motor wall, is required for data collection.

The traditional sensor of a motor monitoring system assembly is shown in Figure 1(b). The sensor is located at the spacer ring of the motor and the signal cable coupled with the power supply is introduced to the motor casing through a hole. These types of system require the same number of signal cables as there is sensor units, yielding a complicated signal wiring system. In addition to the high costs of machining processes, the sensor cable assembly may also cause damage, further lowering the reliability of the motor monitoring system.

The development of wireless and powerless sensing nodes aims to lower costs and increase the reliability of the motor monitoring system as due to its simple wiring, the deployment of the novel-sensing node offers low costs. The performance of the monitoring system and its ability to improve system reliability was investigated in this study. Since fault signatures usually originate from abnormal vibrations the vibration sensors demand higher data transfer rates than other temperature, or pressure, sensors that are employed for motor monitoring, therefore, this study focused on vibration measurements.

### 2.2. MEMS accelerometer

A MEMS (Micro Electro-Mechanical System) sensor was employed for wireless and powerless sensing nodes. Compared with the piezoelectric accelerometer employed by traditional monitoring systems, the MEMS accelerometer is silicon micro-machined, and therefore, can be easily integrated with the signal processing circuits. Moreover, the power consumption of the MEMS accelerometer has clear specification, as provided by the manufacturer, facilitating the self-powered generator design. The commercial product, Kionix KXPA4 series accelerometer [11], was selected for this study.

Specifications and their comparisons to the traditional piezoelectric accelerometer, namely the KISTLER type 8636C5, are listed in Table 1. With the exception of the lower resonant frequency, the MEMS accelerometer has similar performance to the piezoelectric system. Note the listed details of power supply demands of the MEMS accelerometer including voltage, current, sleep modes, and wake-up times of power consumption, which can be useful information for self-powered generator designs. The piezoelectric accelerometer sensor operations rely on the power supply of the front-end amplifier, which is connected directly to the analysis instrument. No power supply information can be referred.

The MEMS accelerometer element functions on the principle of the differential capacitance of the micro machined structure of the silicon chip. Acceleration causes the displacement of a silicon structure resulting in capacitance changes, which can provide tri-axial vibration detection of a variety of upper frequencies. In this study, x-axis with 3.3 kHz resonant frequency was employed for motor radial direction vibration measurements.

The chip embedded signal processor, using a standard CMOS manufacturing process, detects and transforms changes of capacitance into an analog output voltage, which is proportional to acceleration. An 8051-core microprocessor, with a build-in AD converter, was employed to track analog input from the sensor and determine the mean value of the acceleration.

### 2.3. EM pulse communication modules and the wireless signal transfer through metal

The acceleration signal from the MEMS accelerometer is analyzed and encoded by the signal processor, which consists of an 8051-core microprocessor and its related circuits. The transceiver of the EM pulse communication module converts the digitized measurement data into sequential electronic pulses, and then transmits to the receiver, which is outside the motor. As shown in Figure 1(a), one signal cable, attached to the motor's surface, acted as a receiver antenna to collect EM pulses. The receiver decoded the high/low duty of the pulses in order to decode the measurement data and sent it to the data center through a serial communication port. The receiver has a voltage gain of 22 dB, meaning the signal can be recovered, without distortion, under a transmission loss of 22 dB. The EM pulse communication module was designed to achieve wireless signal transfer through the media of metal, not air. The special transmission path needs to be analyzed in detail to obtain the estimated power consumption for the transceiver design.

This semi-analytical approach [12], the *A−φ* method, was proposed to analyze wireless transfer through metal casing. Based on the governing Maxwell equations:

(1) $$\nabla \times E=-\frac{\partial B}{\partial t}$$

(2) $$\nabla \times H=J+\frac{\partial D}{\partial t}$$

(3) ∇⋅D=ρ

(4) ∇⋅B=0

(5) $$\nabla \cdot J=-\frac{\partial \rho }{\partial t}$$

where *E* is the electric field intensity, V/m; *D* is the electric flux density, C/m; *H* is the magnetic field intensity, Ampere/m; *B* is the magnetic flux density, T; *J* is the current density, Ampere/m^2^, and ρis the electric charge density, C/m^3^.

Since ∇ ⋅ *B* = 0, we can derive *B* = ∇×*A*

Substitute this into Eq. (1), we can get

(6) ∇×(E+jωA)=0

Eq. (6) can be expressed by

(7) E+jωA=−∇φ

Since the two-parameter model, the method is so called the *A−φ* method.

Since *B* = *μH*, μ is the magnetic permeability, and *B* = ∇×*A* and $\frac{\partial D}{\partial t}=\gamma E$, we can obtain

(8) ∇×∇×A=μJ−jωμγA−μγ∇φ

Using identical equations for the vector, we can obtain

(9) $$\nabla \left(\nabla \cdot A\right)-\nabla^{2}A=\mu J-j\omega \mu \gamma A-\mu \gamma \nabla \varphi$$

Since

(10) $$\nabla \cdot A=\frac{\partial A_{x}}{\partial x}+\frac{\partial A_{y}}{\partial y}+\frac{\partial A_{z}}{\partial z}=0$$

(11) $$\nabla \varphi =\frac{\partial \varphi }{\partial x}\hat{i}+\frac{\partial \varphi }{\partial y}\hat{j}+\frac{\partial \varphi }{\partial z}\hat{k}=0$$

We get

(12) $$\nabla^{2}A=-\mu J-j\omega \mu \gamma A$$

This is a time-dependent partial differential equation. Assuming the pulsed field can be applied along one axis, Eq. (12) becomes a simple conduction problem. We calculated the differential terms on both sides of the equation by point finite difference scheme and the dissipation term, *μJ*, can be determined. This term indicates the power dissipated during signal delivery and the transceiver power consumption of the EM pulse communication module can be estimated. With respect to different pulse ranging in duration from 0.1 to 1 msec, the power dissipations for data communication were estimated to consume 4.4 to 0.41 mW. The overall power consumption of the EM pulse communication modules is listed in Table 2.

### 2.4. Sensor node power consumption analysis

The sensor node relies on the power supply from the self-powered generator. The power consumption of each component of the sensor node was analyzed in detail to ensure the generator could provide sufficient capacity. The generated amount of electric power not only supplies the nominal works of the circuits, but also meets with the demands of high workload periods.

Table 2 lists the power consumption of each component of the wireless and powerless sensor node, and by referring to datasheet [11], the MEMS accelerometer nominal working power can be determined. The signal processor with an 8051 core requires different power consumptions under signal counting, data encoding, and communication stages. The nominal working power requirements were tested under data streaming simulation conditions and maximum power was determined by the current limits of the microprocessor. Based on analysis of the model described in the above section, the EM pulse communication module power consumption was estimated according to motor casing geometry. Since the wireless signal transfers through material is the unusual working condition, the demands for power by the EM pulse communication module is estimated, and therefore, the value has a varying span due to analysis uncertainties.

### 2.5. Self-powered generator design

**Figure 2.**
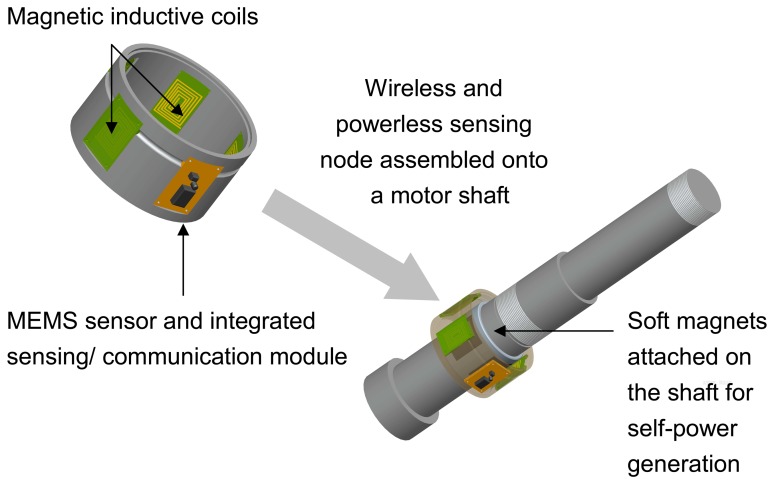
The magnetic self-power generator design and the integration with the wireless and powerless sensing node

The self-power generator consists of coils; recertify circuits, and a magnetic ring, which generates power through electromagnetic induction. The soft magnet ring is attached on the shaft and induces a current through a coil when the motor is started; the alternate current was rectified to provide power for the sensor node. Due to its ease of integration, a planar coil was used in the design system. Figure 2 shows the magnetic self-powered generator design and integration of the wireless, and powerless, sensing node.

For a given power, P, and the working current, I, the number of turns of the planar coil N can be calculated by the following equation [13, 14]

(13) $$N=\frac{1}{I}\sqrt{P\frac{h}{2\pi \rho_{w}}\frac{R_{\max}-R_{\min}}{R_{\max}+R_{\min}}}$$

Where h is the height of the wire; ρ_w_ is the electrical charge density of the wiring material; R_max_ and R_min_ are the outer and inner coil radius, respectively.

With respect to the magnetic self-powered generator, as proposed in this study, power P was targeted to meet the sensor node power demands, as analyzed in the previous section, the current I should meet the maximum demands listed in Table 2. The value of h depends on the coil wire gauge. The electric charge density, ρ_w_, can be calculated by the Faraday induction law by the input of the shaft rotation speed. R_max_ was limited by the size of the sensing node. Through the input of the above parameters, the number of turns of the planar coil N can be determined by the iterations, and thus, the magnetic self-powered generator design can be completed.

### 2.6. Signal processing of sensor node data

The wireless and powerless sensing node sends measured data by EM pulses. The transceiver of the EM pulse communication module converts the digitized data into sequential pulses, and then transmits to the receiver. Non return to zero (NRZ) pulse-code modulation is employed for an 8-bit representation of the time domain signal. The differential modulation algorithm is used to enhance communication speed. Data at sampling rate up to 10 kHz can be delivered by the EM pulse of 0.1 msec width. The receiver acquires sequential pulses and decoded the pulses into a time-domain vibration signal. Digital filters process digitized signals to reject noise. For vibration measurements, a Hanning window function was applied and the anti-aliasing filter would pass all frequencies up to half of sampling frequency. The 512-point fast Fourier transform (FFT) processor is used to generate the power spectrum for identification of vibration sources.

## 3. Experiments

**Figure 3.**
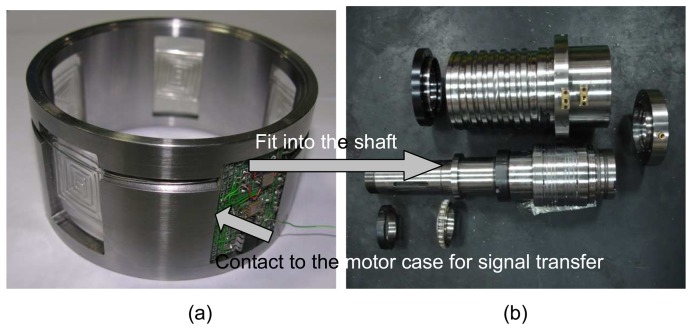
Photographs of the wireless and powerless sensing node (a) and the assembly to the spindle motor shaft (b)

The prototype of the wireless and powerless sensing node was constructed and assembled into a spindle motor for tests. Figure 3 shows photographs of the assembly processes for the sensor node. This study focused on vibration measurement applications using a sensor node equipped with an MEMS accelerometer. Experiments were conducted to compare the frequency responses between the MEMS accelerometer and the piezoelectric system. Two features of the sensing node are its wireless signal transfer through metal and ease of wiring installation. A special EM pulse communication module was built to meet any special demands. Communication status and signal transmission distances were tested in the experiments. The main purpose of this study is to enhance the reliability of the monitoring system through the development of a novel-sensing node. A fault-simulated experiment was conducted to test this monitoring system, equipped with a wireless and powerless sensing node against a traditional system using a sensor with a connection line.

### 3.1. Accelerometer performance test

The frequency responses of the MEMS accelerometer and piezoelectric system were measured using swept sine excitation [15]. The vibration amplitude was controlled through acceleration; the experiment ran a swept sine between 10 to 8 kHz, controlled with 1g acceleration. The sweeps went in only one direction and the frequencies were linearly varied. Accelerometers were mounted to a vibration table and connected to a FFT spectrometer to record the data. Throughout this test, the accelerometer's practical resonant frequency can be determined and the signal output with respect to different vibration frequencies, can be examined.

### 3.2. Communication module performance test

The main feature of the wireless and powerless sensor node is the wireless signal's ability to transfer through a special media, metal casing. The EM pulses were employed to deliver sensor signals from inside the motor. Based on the electromagnetic field analysis, the EM pulse communication module was designed and fabricated. The sensor node was assembled into a spindle motor to measure the signals of vibration, which are caused by motor rotation. The communication performance of the module was tested by measuring motor vibration with a rotation speed of 6,000 rpm. Vibration peak frequency of at least 10 dB signal to noise ratio can be identified through the rotation speed times the rolling elements of the bearings and occurs at approximately 2.4 kHz; the module tested should be able to identify this frequency. Additionally, the transfer distance of the wireless signal was investigated by changing the location of the receiver signal cable away from the sensor node communication contact point. The communication distance issue is critical to this study. Many sensor node applications may be distributed in a machine, data acquisition relies only on one or two signal lines. The farther the distance that communication are able to reach, the lower the construction costs for the monitoring system equipped with the novel sensor nodes can be achieved.

### 3.3. Reliability tests of motor monitoring system

The main purpose of the wireless and powerless sensor node development is to enhance the reliability of motor monitoring systems. In the harsh environment of industrial plants, the sensor wiring assembly can be damaged rendering the deployed monitoring system to become unreliable. Using this special wireless signal transfer system that is able to transfer through metal complicated sensor wiring can be avoided and a simple and reliable motor monitoring system is obtained. The fault-simulating experiments were set up to test the reliability of traditional systems against one equipped with the novel-sensing node. Under testing conditions where the signal cable was bent, lengthened, and even pulled apart, this motor monitoring system measured the vibrations caused by motor rotations, similar to those described in the above section, and then data transferred were tested. Through the test results, the systems resistivity to common fault conditions: signal cable sustaining material damaged was investigated and the reliability of the monitoring system equipped with a wireless and powerless sensing node was discussed.

## 4. Results and Discussion

**Figure 4.**
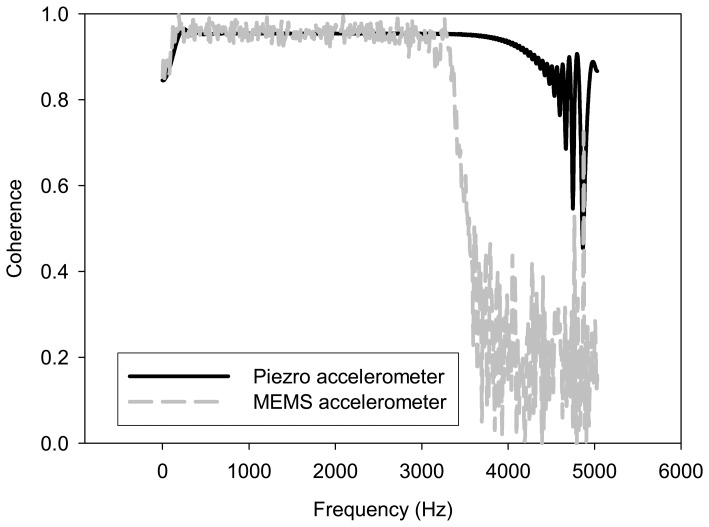
Frequency responses of a MEMS accelerometer and a piezoelectric accelerometer

A motor monitoring system equipped with a wireless and powerless sensor node was constructed as shown in Figure 3. Since fault signatures usually come from abnormal vibrations, the vibration signal demands a higher data transfer rate than other temperature or pressure sensors can provide, this study focused on measuring vibrations using a MEMS accelerometer. The experiment was set up to test frequency responses of the MEMS accelerometer. Its performance was compared with the piezoelectric accelerometer, which is employed by traditional motor monitoring systems. Figure 4 shows the test results. Due to the resonant frequency limitations, as described in Table 1, the MEMS accelerometer cannot detect high frequency vibration with a flat response. Comparing with the flat response provided by the piezoelectric accelerometer of up to 5 kHz, the upper frequency limit of the MEMS accelerometer is 3 kHz. However, it performed well for the experiments in this study because the test motor was running at 6000 rpm and the vibration frequency was expected lower than 3 kHz.

The sensing node was assembled into a 2.2 kW spindle motor and the working status was investigated. According to the power consumption analysis, shown in Table 2, the sensing node works independently with no interference to the motor because the sensing nodes power is small relative to the motor. Under real test conditions, the sensor node inside the motor continuously sent MEMS accelerometer signals to the outside receiver by EM pulses when the motor started.

**Figure 5.**
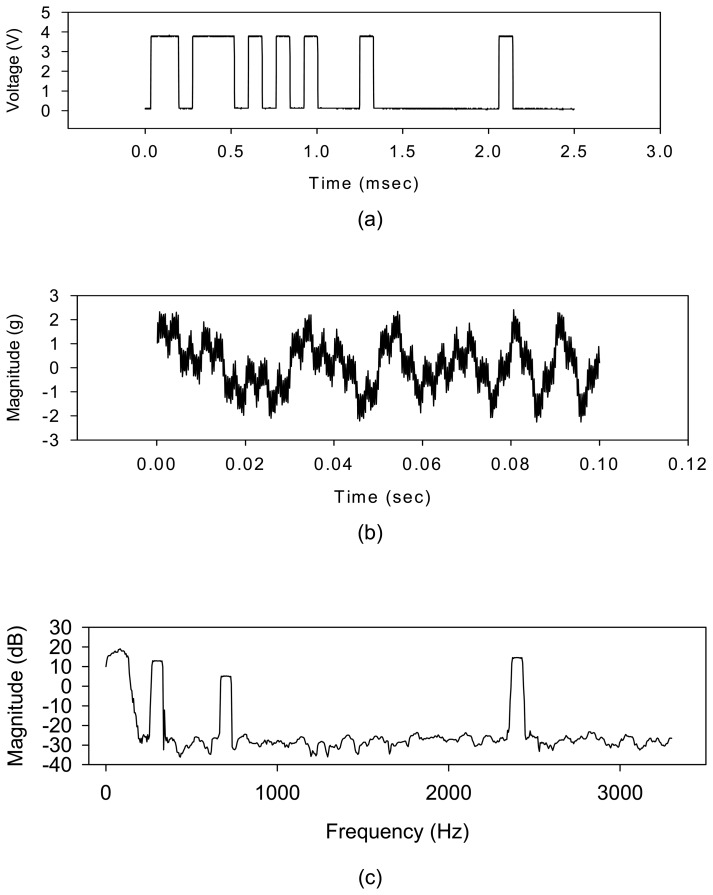
The vibration measurement output of the wireless and powerless sensor node: **(a)** Encoded EM pulses from the transceiver **(b)** Time domain waveform decoded by the receiver **(c)** FFT analysis result

Figure 5(a) shows encoded EM pulses from the transceiver. The outside receiver decoded the pulses into a time-domain signal with a resolution of 0.016 msec. Figure 5(b) shows the waveform of the vibration signals. A 512-point FFT was employed to analyze the time domain signal and the vibration characteristics of the motor were identified. Figure 5(c) shows the spectrum. The motor rotation speed is 6000 rpm. The 100 Hz and 300 Hz peak of the spectrum shown on Figure 5(c) could be identified as shaft rotational frequency and its third harmonic. An 800 Hz peak was identified as the rolling element bearing frequency. The amplitude of its third harmonic at frequency 2.4 kHz was uncommonly higher than the bearing peak. That may be caused by the assembly's unbalance. The results indicated the effective works of the wireless and powerless sensor node because the vibration characteristics were correctly identified. The sensor node employs four coils to generate power and the interference to the motor shaft should occur at a frequency equals to four times the rotational frequency. However, no 400 Hz peak was observed in the spectrum. That illustrates the power generation of the sensor node had no influence upon motor rotating. The wireless and powerless sensor node works in a self-sustainable status, therefore, the feasibility of the wireless signal transfer through metal casing is proved.

**Figure 6.**
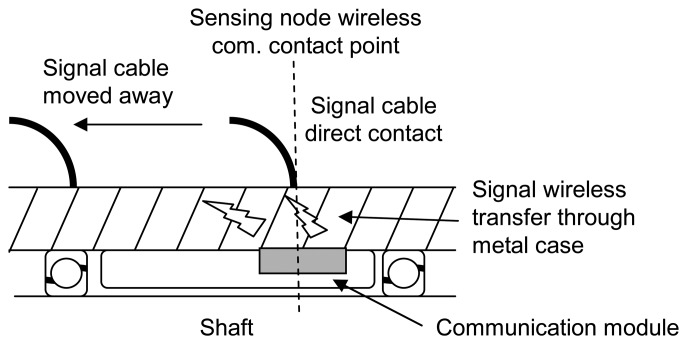

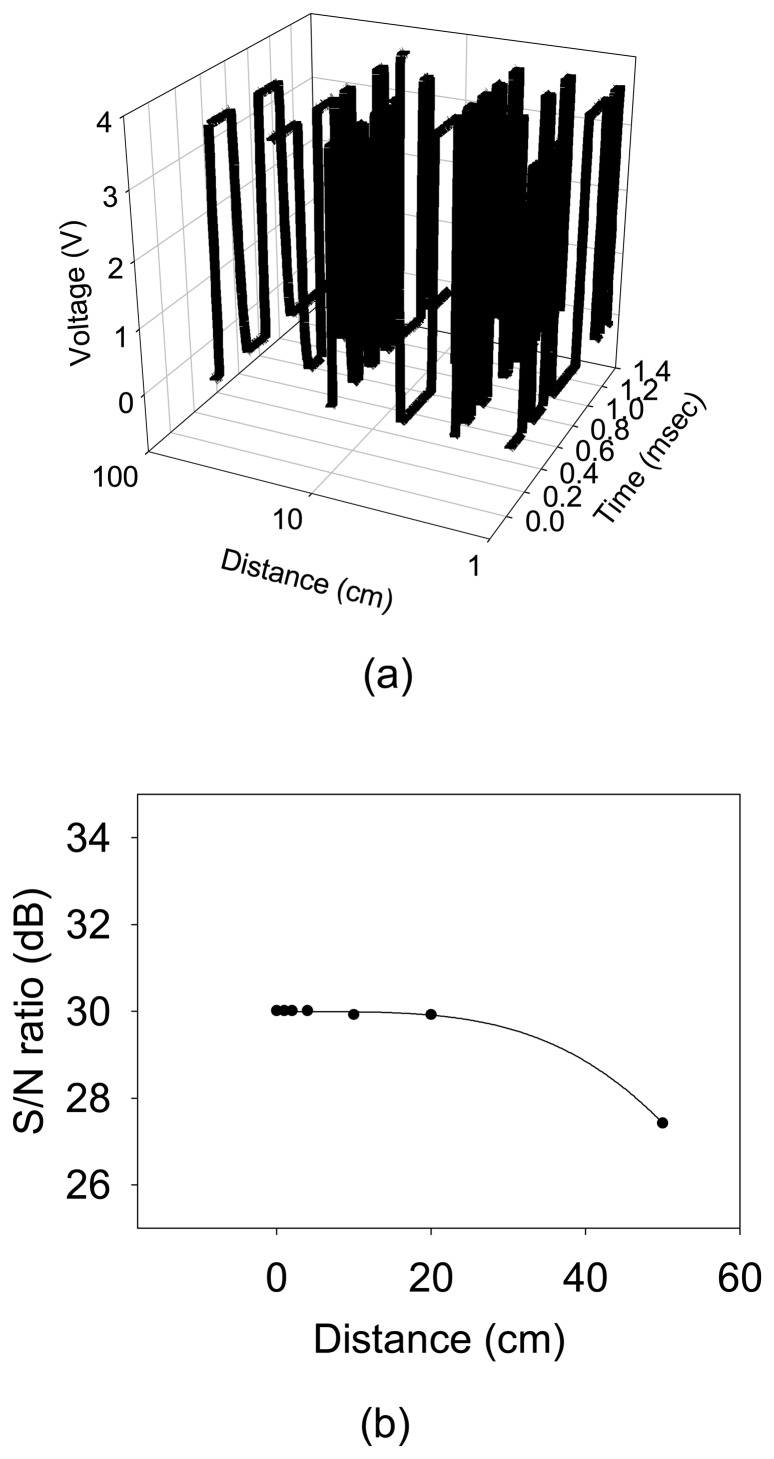
The signal outputs of the wireless and powerless sensor node with respect to different distances between the signal cable point of attachment and the sensor node's wireless communication contact point (a) the signal to noise ratio changes with the distances (b)

The distance of the special wireless signal transfer through metal was an issue. For the spindle motor with a 40 mm wall thickness, we tested the signal transfer under the conditions of the signal cable attached directly to the metal casing, positioned above the sensing node assembly's position, and then sequentially moved it to 1 cm, 2 cm, 5 cm, 10 cm, 20 cm, and 50 cm. Due to size limitations, the longest test distance was 50 cm, as shown in Figure 6(a), the EM pulse output of the sensor node. The signal to noise ratio of different communication distances were calculated, Figure 6(b) shows the results. The experiment observed less than 3 dB decay with respect to 50 cm communication distance, the same complete signal can be obtained by the 22 dB voltage gain receiver. The results illustrates that, one signal cable can be used to gather the signal from the distributed sensor nodes, in a spindle motor, within 1 meter axial range. The simplicity of the signal wiring is of great help when building a reliable motor monitoring system.

**Figure 7.**
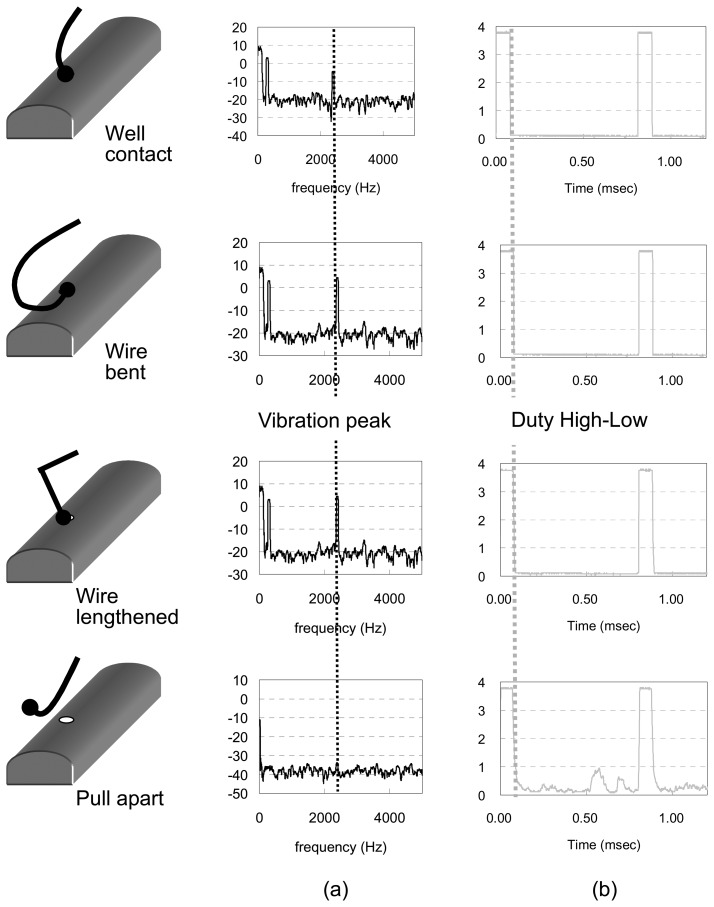
The signal outputs of (a) wireless and powerless sensing node and (b) traditional system sensor under different conditions that the connection wire to data acquisition center was bent, lengthened, and even pulled apart

The fault-stimulated experiments were set up to test the reliability of the monitoring system equipped with a wireless and powerless sensor node. Under test conditions were the signal cable was bent, lengthened and even pulled apart, the motor monitoring system detected the vibration of the motor and measured signals were sent back. These measurement results were compared with those obtained through a traditional system. Figure 7 shows the comparison of the signal outputs of a sensor node and the traditional sensor system. The traditional system, equipped with a piezoelectric accelerometer gave vibration spectra, as shown in Figure 7(a). The identified peaks had different amplitude, with respect to the same motor rotation, under test conditions where the signal cable was bent or lengthened due to the mechanical link through the cable line and vibration measurements were interfered with by external forces. As the signal line was pulled apart by a large force, the traditional system lost the signal. For the system equipped with the sensing node, able to transfer wireless signals, the external force did not act directly on the sensor, and therefore, had almost no influence on the motor vibration measurements. Figure 7(b) gives the EM pulse outputs of the sensor node under different test conditions. Almost the same high low duty waveforms with respect to cable line bends or lengthening conditions, as shown on the figure, illustrates the same vibration spectra can be obtained. It is noticed that the EM pulses can still maintain data transfer even after the signal line was pull apart. Under this condition, the low duty voltage of the EM pulse was fluctuated. However, the vibration signal still can be recovered by the receiver.

**Figure 8.**
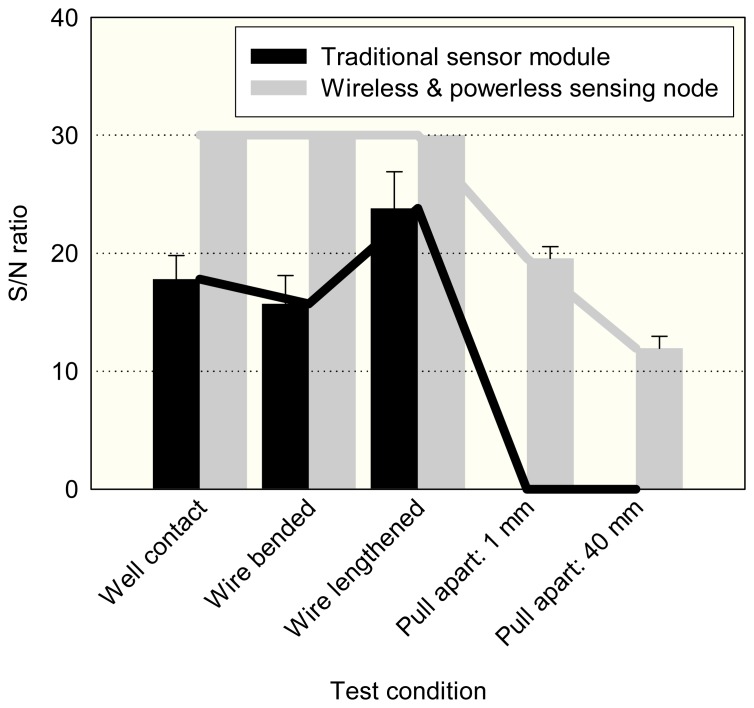
The signal to noise ratios of the wireless and powerless sensing node and the traditional system sensor outputs under the different conditions that the connection wire to data acquisition center was bent, lengthened, and even pulled apart

Figure 8 shows the signal to noise ratios of two monitoring systems with respect to the different test conditions. For the traditional system, the significant signal to noise ratios changed, ranging from 24 dB to 17 dB, then the signal was totally lost as the cable was pulled apart. For the system equipped with the sensor node, the vibration measurements had 30 dB signal to noise ratios without any changes when test conditions bent, or lengthened, the cable. Even when the signal cable was pulled apart at 40 mm, the EM pulses could penetrate through the air gap and the data acquisition center consistently gathered vibration measurement results under a 12 dB signal to noise ratio. The results show that the wireless and powerless sensor node provides a fault tolerant solution to motor monitoring systems.

## 5. Conclusions

A wireless and powerless sensor node consisting of a MEMS accelerometer, a signal processor, a communication module, and a magnetic self-powered generator was developed in this study. The feasibility of data transfer from sensors inside a machine to an outside data center was that the transmission through the metal casing does not require any signal cable and power line connection. The motor monitoring system equipped with the novel-sensing node was constructed and tested. The proposed power mining technique uses the rotational motion of a motor to generate sufficient power for enabling the sensor node. It can provide a consistent sensor signal output with a high S/N ratio even under the worst case where the signal cable was pulled apart. This illustrates that this novel sensor node development can effectively enhance the reliability of a motor monitoring system.

A special wireless signal transfer was reported in this study. Unlike the usual concept, where the wireless communication is through the air; this study demonstrated a wireless signal transfer through a metal casing. Using an EM pulse, the signal can be transmitted from the inner location of a machine to the outer wall for data acquisition. A related electromagnetic field was analyzed to obtain the design principles of the transceiver and receiver. A special communication module was fabricated to transmit the signals of a MEMS accelerometer and the applicability of the proposed structure is verified by the experimental results. Since the vibration sensor demands higher data transfer rates than other temperature or pressure sensors employed for motor monitoring, the success of vibration signal transmission means the EM pulse communication can be applied to the signal transmissions of all types of sensors.

A new type of WSN was proposed by this study. The sensors distributed inside the machine can be linked by WSN architecture through wireless signals transferring through metal casing, which can eliminate the costly installations of communication cables. The deployment of WSN results in a sensor-rich environment able to construct an intelligent and high-level management system for tooling machines or other advanced industrial applications.

## Figures and Tables

**Table 1.** Comparison of the MEMS accelerometer and piezoelectric accelerometer

**Parameters**	**Units**	**MEMS accelerometer Kionix KXPA4series**	**Piezoelectric accelerometer KISTLER type 8732A500 Serial No. 2036346**

Range	g	±6	±5
Sensitivity	mV/g	560	20
Transverse sensitivity	%	2.0	1.0
Operating temperature	°C	-40 ∼ 85	-50 ∼ 120
Resonant frequency	kHz	3.3 (X, Y axis) 1.7 (Z axis)	9.0
Power supply	V	2.8 V
	mA	1.1 (Typical)	NA
	μA	< 10 (Shutdown pin connected)	
	ms	1.6 (Power up time)	

**Table 2.** The power consumptions of each component of the wireless and powerless sensing node and the design target for a magnetic self-powered generator

Item	Power supply	

MEMS accelerometer	2.8 V	Power consumption:
	1.1 mA	3.08 mW
Signal processor	4 V	Nominal power consumption: 21.6 mW
	1.8 ∼ 9 mA	Transient power max: 36 mW
	4 V	10 mW processing power+ 0.41 ∼ 4.4
EM pulse communication module	2.6 ∼ 3.6 mA	mW power dissipations estimated by the method described in section 2.3.

Total		35.08 mW (Nominal)
		53.48 mW (Max)

Max Current		13.7 mA
Power supply design target		53.48 mW(Max) × 2 (S.F)= 107 mW
